# Therapeutic plasma exchange does not reduce vasopressor requirement in severe acute liver failure: a retrospective case series

**DOI:** 10.1186/s12871-015-0017-9

**Published:** 2015-03-08

**Authors:** Ubbo F Wiersema, Susan W Kim, David Roxby, Andrew Holt

**Affiliations:** 1 Department of Critical Care Medicine, Flinders Medical Centre, Bedford Park, South Australia Australia; 2 Flinders Centre for Epidemiology and Biostatistics, School of Medicine, Flinders University, Bedford Park, South Australia Australia; 3 SA Pathology Transfusion Service, Flinders Medical Centre, Bedford Park, South Australia Australia

**Keywords:** Acute liver failure, Therapeutic plasma exchange, Plasmapheresis, Vasopressor, Shock, Haemodynamic

## Abstract

**Background:**

In acute liver failure (ALF) therapeutic plasma exchange (TPE) improves laboratory measures of liver function. In patients with ALF requiring minimal vasoactive support TPE has also been shown to provide haemodynamic benefits including an increase in systemic blood pressure. However the haemodynamic effects of TPE in patients with severe ALF requiring moderate or high dose vasopressor therapy has not been reported. We retrospectively examined the haemodynamic effects of TPE in a cohort of patients with severe ALF requiring vasopressor therapy.

**Methods:**

Physiological, laboratory and treatment data were collected on all patients with ALF who received TPE between January 2000 and December 2012. All patients were managed in the intensive care unit of a tertiary referral centre for ALF and liver transplantation.

The primary outcome measures were changes in mean arterial pressure (MAP), vasopressor score and the ratio of vasopressor score to MAP (vasopressor dependency index (VDI)) from baseline prior to TPE through to 12 hours after completion of TPE. Secondary outcome measures were changes in other routinely collected physiological variables and laboratory results. Results are presented as median (interquartile range (IQR)). Outcome measures were evaluated using a mixed effect model.

**Results:**

Thirty nine TPE were performed in 17 patients with ALF (13 paracetamol poisoning). All TPE were performed with a centrifugal apheresis system (duration 130 minutes (IQR 115 – 147.5), plasma volume removed 5.1% body weight (IQR 4.6 – 5.5). Baseline values for primary outcome measures were: MAP 82 mmHg (IQR 72 – 92.5), vasopressor score 8.35 (IQR 3.62 – 24.6) and VDI 0.10 (IQR 0.05 – 0.31).

MAP was significantly higher immediately after TPE compared to baseline (*p* = 0.039), however when corrected for change in vasopressor requirement there was no significant change in VDI with TPE (*p* = 0.953). Twelve hours after TPE the MAP, vasopressor score and VDI were not significantly different from baseline (*p* = 0.563, *p* = 0.317 and *p* = 0.214 respectively).

**Conclusion:**

In this cohort of patients with severe ALF centrifugal TPE did not significantly affect vasopressor requirements.

**Electronic supplementary material:**

The online version of this article (doi:10.1186/s12871-015-0017-9) contains supplementary material, which is available to authorized users.

## Background

Acute liver failure (ALF) is defined as the rapid onset of encephalopathy and coagulopathy after the onset of jaundice in patients with no known previous liver disease [1]. In severe cases multiple organ systems are affected, but with no known curative therapy, treatment is aimed at providing physiological support and preventing irreversible intracranial hypertension until, either a suitable cadaveric liver becomes available for transplantation, or spontaneous recovery of liver function occurs [2,3]. Prolonged supportive therapy may be necessary in regions with a small potential donor pool, or where transplantation is contraindicated. A central component of supportive care is vasopressor therapy for distributive shock.

In the setting of ALF therapeutic plasma exchange (TPE) with fresh frozen plasma as the replacement fluid improves laboratory measures of coagulation [4-7], facilitating invasive procedures, without the risk of excessive fluid loading. Furthermore liver enzyme and ammonia levels are reduced [5-8], but whether there is an improvement in outcome (transplant free survival) remains uncertain [3,9]. Larsen and colleagues demonstrated potentially beneficial haemodynamic effects of TPE in ALF, with an increase in blood pressure and reduction in cardiac output without a change in oxygen consumption [10,11]. However, these results were obtained in a small series of patients requiring little or no vasoactive therapy (dopamine or dobutamine only).

At our institution TPE is used in selected patients with severe ALF most of whom require moderate or high dose vasopressor therapy to maintain a clinically acceptable blood pressure. The primary aim of this study was to retrospectively examine our experience of the haemodynamic effects of TPE in ALF, particularly the temporal effect on vasopressor requirements. Secondarily, we analysed the effect of TPE on respiratory, coagulation and biochemical variables.

## Methods

This retrospective study was conducted in the intensive care unit (ICU) of a university teaching hospital and referral centre for acute liver failure and liver transplantation. Approval for the study was obtained from the Southern Adelaide Clinical Human Research Ethics Committee. Informed consent was waived as data collection and analyses were anonymised.

### Patients and treatment

All patients with a diagnosis of ALF who presented to the hospital between January 2000 and June 2012, and who underwent TPE, were identified from a computerised database for TPE held in the hospital transfusion service. Patient demographic and clinical information was obtained from historical case notes. Physiological and therapeutic data (hourly recordings) were obtained from historical ICU 24 hour flow charts. Laboratory results were obtained from the hospital computerised laboratory results system (Oacis Clinical Care Suite, Dinmar, USA). TPE data were obtained from the hospital transfusion service TPE database. Liver transplant listing data was obtained from the hepatology service liver transplant database.

All patients underwent TPE with a Spectra Optia apheresis system (Terumo BCT Inc, Lakewood, Colorado, USA). Citrate based anticoagulation was used with every TPE. Intravenous calcium therapy was given as required for hypocalcaemia. All treatments were performed in the ICU. All patients had invasive arterial and venous blood pressure monitoring in situ.

For each patient demographic data were collected for age, weight, cause of acute liver failure, time from admission to ICU until first TPE treatment, liver transplant listing, liver transplantation, severity of illness (APACHE III and SAPS II), treatment with N-acetylcysteine, renal replacement therapy, use of intracranial pressure monitoring and mortality.

### Measurements

For each TPE the following treatment variables were collected: start time, finish time, volume processed, volume removed (effective dose of TPE), volume replaced, type of replacement fluid, total acid-citrate-dextrose volume used and acid-citrate-dextrose volume received by patient. Patient physiological and laboratory values were collected at the following time points relative to each TPE: within 1 hour before treatment (pre-treatment), each hour during treatment, and at 1, 2, 3, 6 and 12 hours after TPE completed. The following physiological and laboratory values were collected at those time points: heart rate, systolic blood pressure, diastolic blood pressure, mean arterial pressure (MAP), central venous pressure, patient temperature, fluid balance (net input and output from a baseline within 1 hour before treatment), vasoactive infusion rates, arterial pH, inspired oxygen concentration (FiO_2_), partial pressure of carbon dioxide (PaCO_2_), partial pressure of oxygen (PaO_2_) and Base Excess, whole blood concentrations of haemoglobin, bicarbonate, sodium, potassium, chloride, glucose, lactate, fibrinogen and ionized calcium, plasma concentrations of albumin and bilirubin, and whole blood International normalised ratio (INR) and activated partial thromboplastin time (APTT).

In view of the use of different vasoactive agents in different patients and at different times the combined dose of vasoactive agents, taking into account their relative potency, is expressed using a vasopressor score. In this study the vasopressor score was calculated as (adrenaline dose × 100 μg/kg/min) + (noradrenaline dose × 100 μg/kg/min) + (vasopressin dose × 10000 units/kg/min), which is a modification of the vasoactive-inotropic score described by Gaies et al. to exclude milrinone [12]. No patients received dopamine or dobutamine. In clinical practice the vasopressor dose is periodically titrated according to blood pressure. Therefore the degree of vasopressor support was also expressed as the vasopressor dependency index (VDI), calculated as the ratio of vasopressor score to MAP [13]; thus the greater the amount of vasopressor therapy the higher the VDI.

### Statistical analysis

Descriptive data are expressed as median with interquartile range (IQR). Statistical analysis was conducted using Stata 13 (StataCorp, Texas, USA) with data expressed as mean ± standard error (SE). Linear mixed effects models were used to estimate the effect of TPE on MAP, vasopressor score and VDI with time (pre-, 1 hour post-treatment (P1) and 12 hours post-treatment (P12)) as fixed effects and each patient and repetition of therapy (between 1 and 6 treatments per patient) nested within each patient as random effects. Other variables that may have affected the response to TPE were included as covariates; these were: pH, lactate and presence or absence of renal replacement therapy just prior to TPE. The final models only included covariates that had *p* values of less than 0.05.

The effect of TPE on heart rate, pulse pressure, central venous pressure, patient temperature, net fluid balance, pH, PaO_2_/FiO_2_, lactate, bilirubin, INR and APTT was estimated using a mixed effect model with the same predictors listed above plus bilirubin. Only variables that were significant at the 10% level in univariate analysis were included for examination in the multivariate model. Therefore the predictors included for each outcome variable are different. Statistical significance was defined as *p* value less than 0.05.

## Results

### Patients

**Figure 1:**
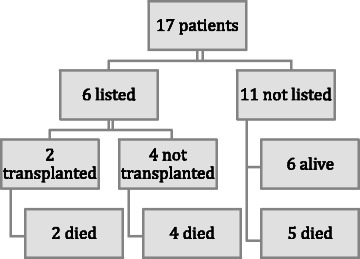
**Patient outcome according to liver transplant status.**

A total of 39 TPE treatments were performed on 17 patients with ALF during the study period; median 2 (interquartile range (IQR) 1–3) TPE treatments per patient. All TPE treatments were included for analysis. The median age was 40 years (IQR 29–45). There were 13 females (76.5%) and 4 males (23.5%). The median weight (IQR) was 60 kg (55–64.5). The median severities of illness (IQR) scores were: APACHE III 107.5 (86–124), SAPS II 55.0 (43.75-66.25). The causes of ALF were: paracetamol toxicity 13 patients, alcoholic hepatitis 2 patients, Budd Chiarri syndrome 1 patient and herbal remedy ingestion 1 patient. Five patients had intracranial pressure monitoring during at least one of their treatments with TPE. There were insufficient data to determine the effect of TPE on intracranial pressure. Patient outcome according to liver transplant status is shown in Figure 1. Six patients survived and 11 patients died.

### Therapeutic plasma exchange

The replacement fluid for the first TPE treatment for every patient was 100% fresh frozen plasma. Additional TPE treatments were performed with 100% fresh frozen plasma for 17 treatments in 13 patients, a combination of fresh frozen plasma and cryoprecipitate for 4 treatments in 4 patients, and a combination of fresh frozen plasma and 4% albumin for 1 treatment. Other TPE treatment variables are shown in Table 1.

**Table 1:** **TPE treatment variables**

Time from admission to ICU to first TPE treatment (hours) median (IQR)	16.67 (2.75-34.67)	
Blood volume processed (ml) median (IQR)	5012 (4373–6147)	
Blood volume processed as % body weight (ml/kg) median (IQR)	8.4 (7.9-9.5)	
Plasma volume removed (ml) median (IQR)	3000 (2689–3390)	
Plasma volume removed as % of body weight (ml/kg) median (IQR)	5.1 (4.6-5.5)	
Citrate dose received by patient (mmol) median (IQR)	27.0 (24.8-31.8)	
Net patient fluid balance during TPE (ml) median (IQR)	267 (−305-433)	
Duration of TPE (minutes) median (IQR)	130 (115–148)	
Number of TPE treatments with renal replacement therapy immediately before and/or after	Before and after TPE	23
	Before TPE only	2
	After TPE only	9
	Neither	5
Ionised Calcium level pre TPE (mmol/L) median (IQR) (laboratory values only available for 34 TPE)	1.05 (0.95-1.16)	
Lowest ionised Calcium level during TPE (mmol/L) median (IQR) (laboratory values only available for 33 TPE)	0.87 (0.71-0.94)	
Ionised Calcium level post TPE (mmol/L) median (IQR) (laboratory values only available for 15 TPE)	1.11 (0.99-1.25)	

### Baseline results

Baseline laboratory results from samples taken before each TPE are shown in Table 2.

**Table 2:** **Baseline laboratory results prior to TPE treatments**

Laboratory test	Pre TPE value median (IQR)
PaO2/FiO2 ratio	248 (166–371)
pH*	7.38 (7.18-7.47)
pH prior to first TPE for each patient*	7.23 (7.07-7.38)
Lactate (mmol/L)	4.7 (2.9-10.5)
Lactate prior to first TPE for each patient	6.3 (4.0-11.7)
Bilirubin (µmol/L)**	80 (54–128)
INR*	2.6 (2.0-4.7)
INR prior to first TPE for each patient*	4.0 (2.2-5.6)
APTT (s)*	50.5 (45.2-58.5)

*One data value missing.

**Six data values missing.

At baseline MAP was significantly higher when the baseline pH was higher (31.9 ± 10.1 mmHg for every unit increase in pH (*p* = 0.002)). Conversely, MAP was inversely related to baseline lactate (0.86 ± 0.37 mmHg lower MAP for every mmol/L increase in lactate (*p* = 0.021)). Vasopressor score and VDI were significantly higher when baseline pH was lower (both *p* < 0.001) and when baseline lactate was higher (both *p* < 0.001). For every unit decrease in pH the vasopressor score was 62.0 ± 14.7 higher and the VDI was 0.82 ± 0.22 units higher. For every mmol/L increase in lactate the vasopressor score was 2.04 ± 0.55 higher and the VDI was 0.04 ± 0.01 units higher. Patients receiving renal replacement therapy just prior to TPE had a higher MAP (*p* = 0.017) and higher vasopressor score (*p* = 0.001) than those not receiving renal replacement therapy, thus there was no significant difference in VDI between the two groups.

### Primary outcome

**Figure 2:**
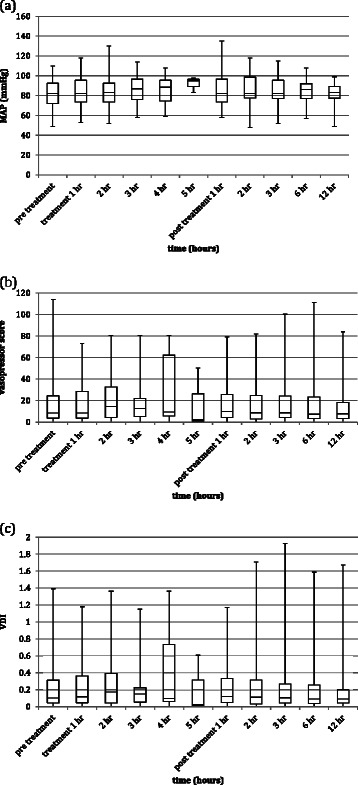
**Box plots for temporal trends in (a) MAP, (b) vasopressor score and (c) VDI for every TPE treatment.** The time points extend from less than 1 hour before TPE (pre treatment), through every hour of TPE (treatment 1 hr, 2 hr etc.) to 12 hours after TPE (post treatment 1 hr, 2 hr etc.). Note that only a few TPE were as long as 4 or 5 hours so the data values for treatment 4 hr and 5 hr are calculated from only a few TPE treatments.

**Figure 3:**
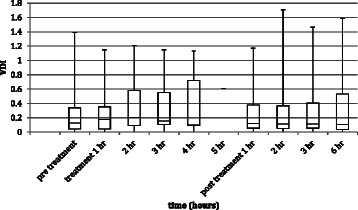
**Box plot for temporal trend in VDI for the first TPE treatment of every patient.** The time points extend from less than 1 hour before TPE (pre treatment), through every hour of TPE (treatment 1 hr, 2 hr etc.) to 12 hours after TPE (post treatment 1 hr, 2 hr etc.). N.B. only a few TPE were as long as 4 or 5 hours so the data values for treatment 4 hr and 5 hr are calculated from only a few TPE treatments.

The temporal trends in MAP, vasopressor score and VDI for every TPE performed are shown in Figure 2. The temporal trends in VDI for the first TPE treatment for each patient are shown in Figure 3. The temporal trends in MAP and vasopressor score for the first TPE treatment for each patient are shown in Additional files 1 and 2. For 7 TPE treatments in 4 patients the patient did not require any vasoactive agent at some (1 TPE) or all time points (6 TPE in 3 patients). The VDI was thus zero for these data points. Physiological variables pre-treatment and 1 hour post-treatment are shown in Table 3. The effect size of TPE on MAP, vasopressor score and VDI, comparing 1 hour post-treatment and 12 hours post-treatment with pre-treatment values and 12 hours post-treatment with 1 hour post-treatment values is shown in Table 4. This demonstrates a significant increase in MAP with treatment (P1 – Pre) (*p* = 0.038), but when corrected for vasopressor dose there was no significant treatment effect on VDI (P1 – Pre) (*p* = 0.953). Twelve hours after TPE the VDI was not significantly different from baseline (*p* = 0.214). No association was found between haemodynamic response and repetition of treatment for the same individual. Analysis of data including only the 13 patients with paracetamol toxicity produced results very similar to analysis of all 17 patients (VDI (P1 – Pre) *p* = 0.865).

**Table 3:** **Physiological values pre-TPE (baseline) and post-TPE (within first hour after treatment)**

Physiological variable	Pre TPE median (IQR)	One hour post TPE median (IQR)
MAP (mmHg)		
All TPE	82 (72–92.5)	82 (73.5-96.5)
First TPE for each patient	82 (72–88)	80 (72–92)
Vasopressor score		
All TPE	8.35 (3.62-24.6)	10.1 (4.30-25.6)
First TPE for each patient	8.58 (3.34-28.4)	10.0 (4.96-34.3)
VDI		
All TPE	0.10 (0.05-0.31)	0.12 (0.05-0.33)
First TPE for each patient	0.13 (0.04-0.34)	0.12 (0.05-0.38)
Heart rate	105 (92.5-111)	112 (92.5-118.5)
Pulse pressure (mmHg)	57 (46.5-69.5)	55 (48.5-71.5)
Central venous pressure (mmHg)	12 (9–16)	11 (9–14)
Patient temperature (Celsius)	36.4 (35.5-37.0)	36.7 (36.1-37.3)
FiO_2_	0.40 (0.35-0.65)	0.45 (0.325-0.55)

**Table 4:** **Treatment effects on MAP, vasopressor score and VDI relative to baseline value** ^**ǂ**^

	VDI		Vasopressor score		MAP	
	Effect size (SE)	P-value	Effect size (SE)	P-value	Effect size (SE)	P-value
**Treatment effect (Pre v P1 v P12)** ^**†**^		0.384		0.557		0.025
P1-Pre	0 (0.04)	0.953	0.32 (2.64)	0.904	3.82 (1.85)	0.039
P12- Pre	0.05 (0.04)	0.214	2.77 (2.77)	0.317	−1.12 (1.94)	0.563
P12-P1	0.05 (0.04)	0.235	2.46 (2.77)	0.375	−4.94 (1.94)	0.011

SE: Standard error.

^ǂ^Adjusted for repeated treatments.

^†^P1-P12: number of hours post treatment.

### Secondary outcomes

The effect of TPE on heart rate, pulse pressure, central venous pressure, patient temperature, PaO_2_/FiO_2_, pH, lactate, INR and bilirubin are shown in Table 5. Although heart rate, patient temperature, bilirubin and INR changed significantly with TPE, none of them remained significantly different from baseline 12 hours later. The temporal trends in heart rate, pulse pressure, central venous pressure, patient temperature and cumulative fluid balance from 1 hour before TPE to 12 hours after TPE are shown in Additional files 1 and 2. There were not sufficient data for fibrinogen to allow meaningful analysis.

**Table 5:** **Baseline measurements and treatment effects on heart rate, pulse pressure, central venous pressure, patient temperature, net fluid balance, pH, PaO**_**2**_ **/FiO**_**2**_ **ratio, lactate, bilirubin, INR and APTT**

	Effect size (SE)	P-value
**Heart rate**		
Treatment effect (Pre v P1 v P12)^†^		0.002
P1-Pre	5.15 (1.88)	0.006
P12-Pre	−1.12 (1.97)	0.568
P12-P1	−6.28 (1.97)	0.001
Baseline lactate	1.4 (0.39)	<0.001
**Pulse pressure (mmHg)**		
Treatment effect (Pre v P1 v P12)		0.456
P1-Pre	0.55 (1.88)	0.772
P12-Pre	2.40 (1.98)	0.226
P12-P1	1.85 (1.98)	0.350
Baseline bilirubin	0.05 (0.01)	<0.001
**Central venous pressure (mmHg)**		
Treatment effect (Pre v P1 v P12)		<0.001
P1-Pre	−1.00 (0.69)	0.146
P12-Pre	−2.96 (0.73)	<0.001
P12-P1	−1.96 (0.73)	0.007
Baseline bilirubin	0.01 (0.004)	0.007
**Temperature (Celsius)**		
Treatment effect (Pre v P1 v P12)		0.011
P1-Pre	0.41 (0.16)	0.013
P12-Pre	−0.04 (0.17)	0.792
P12-P1	−0.45 (0.17)	0.008
**Fluid balance (ml)**		
Treatment effect (Pre v P1 v P12)		0.021
P1-Pre	34.1 (254.1)	0.893
P12-Pre	−627.5 (264.9)	0.018
P12-P1	−661.6 (564.9)	0.013
**pH**		
Treatment effect (Pre v P1 v P12)		0.053
P1-Pre	0.03 (0.02)	0.122
P12-Pre	0.04 (0.02)	0.025
P12-P1	0.01 (0.02)	0.798
**PaO**_**2**_ **/FiO**_**2**_		
Treatment effect (Pre v P1 v P12)		0.073
P1-Pre	−0.05 (0.21)	0.801
P12-Pre	−0.39 (0.18)	0.029
P12-P1	−0.34 (0.22)	0.118
RRT at baseline	−1.40 (0.49)	0.005
**Lactate (mmol/L)**		
Treatment effect (Pre v P1 v P12)		0.588
P1-Pre	0.25 (0.57)	0.664
P12-Pre	−0.35 (0.46)	0.449
P12-P1	−0.60 (0.62)	0.331
**Bilirubin (µmol/L)**		
Treatment effect (Pre v P1 v P12)		0.026
P1-Pre	−44.4 (17.3)	0.010
P12-Pre	5.33 (11.7)	0.649
P12-P1	49.7 (19.3)	0.010
**INR**		
Treatment effect (Pre v P1 v P12)		0.025
P1-Pre	−1.55 (0.63)	0.014
P12-Pre	−0.90 (0.55)	0.103
P12-P1	0.65 (0.75)	0.383
**APTT (s)**		
Treatment effect (Pre v P1 v P12)		0.421
P1-Pre	−1.57 (4.12)	0.703
P12-Pre	−4.53 (3.45)	0.189
P12-P1	−2.97 (4.41)	0.501

SE: Standard error.

† P1-P12: number of hours post treatment.

## Discussion

### Summary of findings

The principal finding of this study is that in a cohort of severely ill patients with ALF, TPE did not significantly affect the amount of vasopressor support required. Although MAP was higher immediately after TPE, the amount of vasopressor support was also higher, with the net result that the ratio of the two (VDI) was unaffected. The same pattern was seen when only the first TPE treatment for each patient was analysed (see Figure 3). Secondary findings of this study were a sustained reduction in INR, a transient reduction in bilirubin, but no significant change in pH, lactate or oxygenation with TPE.

### Comparison with previous studies

ALF is characterised by a hyperdynamic circulation, with low systemic vascular resistance, high cardiac index and low mean arterial pressure [2,3]. In a prospective study of patients with ALF, Larsen and colleagues found a significant increase in MAP, decrease in cardiac index and decrease in systemic vascular resistance during high-volume plasmapheresis (TPE) [10,11]. There are several reasons why our results differed from these findings. Firstly, paracetamol toxicity was the aetiology of ALF in 13 out of 17 patients in our study compared to 5 out of 16 in the Larsen study. Secondly all of our study patients received N-acetylcysteine compared to only 10 out of 16 patients in the Larsen study. In a sub-analysis of their study Larsen and colleagues found no difference in baseline haemodynamic variables or haemodynamic response to TPE between patients who received N-acetylcysteine therapy and those that did not. However, N-acetylcysteine has been reported to increase mean arterial pressure and cardiac output in patients with ALF [14]. TPE may have increased N-acetylcysteine clearance reducing these haemodynamic effects whilst the patients were receiving TPE. Thirdly, it is likely that our study population had more severe vasoplegia than those in Larsen’s study, where only a minority of patients received vasoactive therapy in the form of “low dose” Dopamine (2–5 μg/kg/min) or Dobutamine (4–14 μg/kg/min) [10,11]. However patients in our study had a higher baseline MAP (82 ± 72–92.5 mmHg) than those in Larsen’s study (74 ± 61–110 mmHg) [10], thus if the vasopressor therapy had been targeted to a lower MAP in our patients less vasopressor would have been required. With the small number of patients studied we were unable to identify a link between vasopressor requirement at baseline (as a measure of vasoplegia) and circulatory response to TPE. Fourthly, Larsen and colleagues used the filtration method of TPE with a 0.65 μm pore size filter [10,11], whereas at our institution all TPE was performed using the centrifugal method. Larsen and colleagues postulated that the hyperdynamic circulation in ALF is attributable to a circulating vasodilating substance or “humoral factor”, perhaps endotoxin, with a high molecular weight, or high degree of protein binding, that could be cleared using the filter used for TPE [10,11]. Such a substance (or substances) should also be cleared with centrifugal TPE, however, if the mechanism for vasodilating substance clearance is adsorption rather than filtration [15], haemodynamic changes may occur with filter based TPE, that do not occur with centrifugal TPE despite the high molecular size clearance. Furthermore, the majority of our patients were also treated with renal replacement therapy (continuous veno-venous haemofiltration) before and after TPE, which may have significantly blunted the potential haemodynamic effect of TPE [16,17]. Indeed high volume haemodiafiltration is associated with a rise in blood pressure independent of acid–base state [18]. Interestingly, in ALF, a single session of therapy with the molecular adsorbent recirculating system increases systemic vascular resistance [19,20]; although this effect was not sustained through a second session [20]. Fifthly, the dose of TPE (volume of plasma removed) was less than half that used by Larsen and colleagues [10,11]. It may be that significant circulatory benefit is only achieved with “high-volume” plasma exchange [9].

Citrate anticoagulation is routinely used with TPE. However, citrate accumulation from inadequate hepatic metabolism can lead to low serum ionized calcium and hypotension. Severe hypocalcaemia was prevented during TPE in our patients by administration of intravenous calcium as required. The precise haemodynamic effects of citrate in ALF have not been clearly established [21], although it appeared to be safe in one study of patients with liver failure (mostly ALF) supported by a molecular adsorbent recirculating system [22]. The infusion rate of citrate in that study was approximately a quarter of the infusion rate in our study, although the duration of infusion was much longer (median 20 hours). It is possible that the mild hypocalcaemia observed during TPE in our study offset possible changes in vasopressor support levels.

### Limitations

The principal weakness was the retrospective nature of this study. Thus management was unblinded, laboratory collection times were not standardised and often incomplete, TPE was variable in timing, dose, repetition frequency and fluid balance, and vasoactive therapy and correction of hypocalcaemia were not standardised. The number of patients and total number of TPE performed were small. The study population was relatively non-heterogenous with the majority having paracetamol poisoning and all receiving N-acetylcysteine therapy. Renal replacement therapy was not standardised in timing, dose or frequency. However, the data were collected from a cohort of severely ill patients with multiple organ failure and high risk of death. Furthermore data was collected for up to 12 hours after TPE and for multiple TPE in the same individual.

A mixed effect model was used to explore the association of baseline pH, lactate and prior renal replacement therapy with haemodynamic response to TPE. These were considered the baseline variables most likely to be associated with haemodynamic response to TPE, either through an association between severity of illness and response to TPE (pH and lactate), or blunting the effect of TPE on haemodynamics (renal replacement therapy). Many other physiological and treatment factors may also have influenced the response to TPE. In this retrospective study these factors could not be controlled for.

Power analysis reveals that the study was underpowered to detect significant changes in the primary outcomes. Using a simulation method for the mixed effects model with effect size and variance of random effects and residuals estimated from the model, at 5% significance level with 80% power, 180 TPE treatment cases would be needed to detect 0.05 unit change in VDI (i.e. 0.27 to 0.32, 18.5% increase in VDI) in 12 hours post treatment from the baseline and at least 300 cases to detect a 3 unit change in vasopressor score in 12 hours post treatment from the baseline. Despite these calculations a significant effect of TPE on MAP was detected, although, when corrected for vasopressor score there was no significant difference in VDI.

### Implications of study findings

The results of this study question the view that TPE improves systemic haemodynamics in patients with ALF. However, the single centre, retrospective nature of this study and small number of patients analysed means that our findings need to be confirmed in future studies. It is unclear whether the differences in our results compared to previous studies are due to differences in patient characteristics (cause of ALF), other aspects of management (N-acetylcysteine therapy, renal replacement therapy), severity of illness (vasopressor requirements) or differences in conduct of TPE such as dose, method (centrifugal versus filtration) or method of anticoagulation. Each of these issues warrant further investigation.

## Conclusion

In a single centre series of severely ill patients with ALF, mostly due to paracetamol poisoning and receiving renal replacement therapy, TPE did not provide a reduction in vasopressor requirement. The results of this study question the use of TPE to improve systemic haemodynamics in patients with ALF.

### Key messages

- In this small retrospective series of patients with acute liver failure, therapeutic plasma exchange did not result in a reduction in vasopressor requirement.
- The findings of this study challenge the view that therapeutic plasma exchange provides haemodynamic benefit in patients with acute liver failure.
- Differences in patient characteristics, or dose and method of plasma exchange (centrifugal versus filtration), may explain the difference in findings between the present study and previous studies.

## Additional files

Additional file 1: Figure S1. Box plots for temporal trends in (a) MAP and (b) vasopressor score for the first TPE treatment for every patient. Time points extend from less than 1 hour before TPE (pre treatment), through every hour of TPE (treatment 1 hr, 2 hr etc) to 12 hours after TPE (post treatment 1 hr, 2hr etc). Note that only a few TPE were as long as 4 or 5 hours so the data values for treatment 4 hr and 5 hr are calculated from only a few TPE treatments. Additional file 2: Figure S2. Box plots for temporal trends in (a) heart rate, (b) pulse pressure, (c) central venous pressure, (d) temperature, (e) FiO_2_ and (f) fluid balance. Time points extend from less than 1 hour before TPE (pre treatment), through every hour of TPE (treatment 1 hr, 2 hr etc) to 12 hours after TPE (post treatment 1 hr, 2hr etc). Note that only a few TPE were as long as 4 or 5 hours so the data values for treatment 4 hr and 5 hr are calculated from only a few TPE treatments.

## Abbreviations

ALF: Acute liver failure
APACHE: Acute physiology and chronic health evaluation
APTT: Activated partial thromboplastin time
INR: International normalised ration
MAP: Mean arterial pressure
SAPS: Simplified acute physiology score
TPE: Therapeutic plasma exchange
VDI: Vasopressor dependency index
