# Supplementary material for: Therapeutic plasma exchange does not reduce vasopressor requirement in severe acute liver failure: a retrospective case series
Source: BMC Anesthesiol. 2015 Mar 8;15:30. doi: 10.1186/s12871-015-0017-9 (PMC4359494; doi:10.1186/s12871-015-0017-9)
Supplement: Additional file 2: Figure S2. — Box plots for temporal trends in (a) heart rate, (b) pulse pressure, (c) central venous pressure, (d) temperature, (e) FiO2 and (f) fluid balance. Time points extend from less than 1 hour before TPE (pre treatment), through every hour of TPE (treatment 1 hr, 2 hr etc) to 12 hours after TPE (post treatment 1 hr, 2hr etc). Note that only a few TPE were as long as 4 or 5 hours so the data values for treatment 4 hr and 5 hr are calculated from only a few TPE treatments. [file 12871_2015_17_MOESM2_ESM.zip › 1381791818146198_add5.docx]

Supplementary figure 2 (b)
